# Carbon Nanotubes Hybrid Hydrogels in Drug Delivery: A Perspective Review

**DOI:** 10.1155/2014/825017

**Published:** 2014-01-21

**Authors:** Giuseppe Cirillo, Silke Hampel, Umile Gianfranco Spizzirri, Ortensia Ilaria Parisi, Nevio Picci, Francesca Iemma

**Affiliations:** ^1^Department of Pharmacy, Health and Nutritional Sciences, University of Calabria, 87036 Arcavacata di Rende, Italy; ^2^Leibniz Institute for Solid State and Materials Research Dresden, Postfatch 270116, 01171 Dresden, Germany

## Abstract

The use of biologics, polymers, silicon materials, carbon materials, and metals has been proposed for the preparation of innovative drug delivery devices. One of the most promising materials in this field are the carbon-nanotubes composites and hybrid materials coupling the advantages of polymers (biocompatibility and biodegradability) with those of carbon nanotubes (cellular uptake, stability, electromagnatic, and magnetic behavior). The applicability of polymer-carbon nanotubes composites in drug delivery, with particular attention to the controlled release by composites hydrogel, is being extensively investigated in the present review.

## 1. Introduction

During the last few years, the development of innovative drug delivery devices for different therapeutic agents has attracted the interest of many researchers. Several studies, indeed, report on the design, synthesis, and characterization of novel materials to be employed as delivery systems in the aim to improve the efficiency of a selected drug [1]. An ideal drug delivery system (DDS) has to maximize the efficacy and the safety of the therapeutic agent, delivering an appropriate amount at a suitable rate and to the most appropriate site in the body. By this strategy, it is possible to prolong the pharmacological activity, reduce the side effects, and minimize the administration frequency resulting in an enhanced patient compliance [2].

The use of innovative approaches in drug delivery field is required by the need of carriers for new biological therapeutic agents, such as nucleic acids and proteins, but also by the pharmaceutical companies due to the upcoming patent expirations and the consequent interest in the development of novel formulations. The employed strategies to achieve alternative drug delivery involve biologics, polymers, silicon-based materials, carbon-based materials, metals, or combinations of them and these materials can be structured in microscale or, more recently, in nanoscale formats [3].

Nanomedicine has been defined as “the monitoring, repair, construction, and control of human biological systems at the molecular level, using engineered nanodevices and nanostructures” [4]. It consists of application of nanotechnology to the diagnosis, prevention, and treatment of diseases and represents a useful instrument to understand specific underlying disease molecular mechanisms [5].

Nanomaterials are promising technologies to detect and diagnose cancer and infectious diseases in their early stages and play a key role in drug discovery, drug delivery, and gene/protein delivery. This kind of innovative materials is able to interact with subcellular structures in the human body with a high specificity, finding a potential clinical application as drug targeting systems in the aim to reduce adverse effects [6].

Nanocarriers are able to release the active compound directly into cells overcoming the biological barriers and discriminating target pathological tissue from the healthy ones [7].

Among the nanotechnologies, polymeric hydrogels have raised a remarkable and considerable interest [8]. Hydrogels represent a class of hydrophilic and crosslinked polymeric networks, insoluble in water and characterized by the ability to hold a large amount of biological fluids, including water, within the spaces available among the polymeric chains. The water holding capacity depends on the presence of hydrophilic functionalities (amido, amino, carboxyl, hydroxyl groups, etc.) in polymer chains and the water content may vary from 10% to thousands of times of the weight of the dry polymer network [9]. Moreover, these polymeric materials present a good biocompatibility and are suitable for applications in medical and pharmaceutical fields including wound dressings, contact lenses, artificial organs, tissue engineering, and drug delivery systems [10, 11].

Hydrogels can be classified according to several parameters, such as their natural or synthetic origin and the cross-linking nature [12].

Generally all polymeric gels are either synthetic (poly (ethylene glycol) (PEG) [13], poly (vinyl alcohol) (PVA) [14], poly (acrylic acid) (PAA) [15], polyacrylamide (PAM) [16], and poly (methyl methacrylate) (PMA) [17]) or natural (alginic acid [18], pectin [19], chitin and chitosan [20], dextran [21], agarose [22], and different derivatives of these polymers).

According to their cross-linking nature, hydrogels can be divided into two main classes: permanent hydrogels, in which covalent bonds between polymeric chains are formed during the cross-linking reaction, and physical hydrogels, in which the cross-linking is due to the formation of physical interactions, such as hydrogen bonding, ionic interaction, van der Waals interactions, and molecular entanglement, among the polymeric chains. Hydrogels based on acrylic monomers are included in the first class, while gelatine and agar-agar hydrogels are examples of materials belonging to the second category [23].

Some of these polymeric networks are able to exhibit phase transition and changes in their equilibrium state in response to external stimuli and are known as “stimuli-responsive” or “smart” hydrogels [24]. This kind of materials finds application in targeted drug delivery due to the capability to respond to environmental factors including physical, chemical, and biochemical stimuli. Physical stimuli consist of temperature, pressure, light, electric, magnetic, and sound fields, while chemical or biochemical stimuli involve pH, ionic strength, ions, or specific molecular recognition events [25]. Stimuli-responsive hydrogels can be classified on the basis of the external stimuli type in thermosensitive, pH sensitive, and electrosensitive [26], as well as sensitive to light, pressure, ionic strength, and different molecules, for example, enzymes and so forth [27]. Different strategies have been developed to apply the external stimuli to the hydrogels, and the main approaches involve the use of suitable buffered saline (phosphate or potassium) for pH responsive materials the increase of the temperature by hot plate and/or IR irradiation in the case thermo-responsive elements and the application of a voltage by appropriate inert electrodes for electroresponsive materials. In the latter case, it is possible to study the response of the hydrogel by modulating the voltage as well as the intensity of the current. Specific example of these matters have been treated below in this review.

## 2. Carbon Nanotubes

Carbon nanotubes (CNTs) are huge cylindrical large molecules consisting of a hexagonal arrangement of sp^2^ hybridized carbon atoms (C–C distance is about 1.4 $\overset{´}{Å}$) [28, 29].

**Figure 1:**
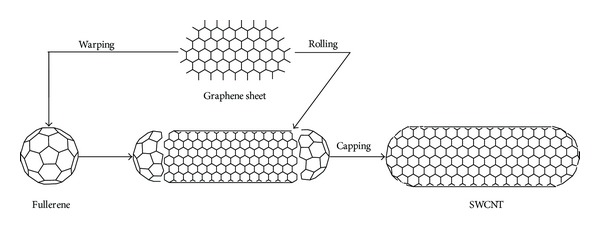
Molecular structures of SWCNT and MWCNT.

The wall of CNTs is composed of one or multiple layers of graphene sheets; thus, it is possible to discriminate these materials in single-walled carbon nanotubes (SWCNT), formed by rolling up of a single graphene sheet, and multiwalled CNTs (MWCNTs), formed by rolling up of more than one graphene sheet. Both SWCNTs and MWCNTs are capped at both ends of the tubes in a hemispherical arrangement of carbon networks called fullerenes warped up by the graphene sheet (Figure 1). The interlayer separation of the graphene layers of MWCNTs is approximately 0.34 nm in average, each one forming an individual tube and the outer diameter ranging from 2.5 to 100 nm, while for SWCNTs this value ranges from 0.6 to 2.4 nm. SWCNTs are characterized by a better defined wall and a smaller diameter that make them suitable as drug carriers due to their quality control. On the contrary, MWCNTs may present defects in their nanostructure resulting in a lack of stability that makes their modification easier [30].

The adopted synthetic procedure influences CNTs length and diameter and, due to attractive van der Waals forces, SWCNTs and MWCNTs tend to pack together in ropes. The obtained bundles contain many nanotubes and are longer and wider than the original ones from which they are formed. This phenomenon could be important from a toxicological point of view [31, 32].

In the past few years, carbon nanotubes have found applications in different fields including nanoelectronics and nanocomposite, due to their considerable electrical, mechanical, and chemical stability, but only in the recent period the interest was focused on their potential application as biomedical devices [33, 34].

Several research studies, indeed, focuses on the use of carbon nanotubes in biological systems [35, 36]. Balavoine et al. used MWNTs for helical crystallization of protein [37], Mattson et al. reported on the first application of carbon nanotube technology to neuroscience research developing new approaches for the growth of embryonic rat-brain neurons on MWNTs [38], and Sadler and coworkers immobilized biological species on both SWNTs and MWNTs for potential biosensor and bioreactor systems [39].

The employment of CNTs as drug excipients with low toxicity and immunogenicity is also largely investigated [40]. The ability of carbon nanotubes to release drugs was studied in the aim to obtain innovative drug vehicles to be applied in the field of nanomedicine [41].

Different types of therapeutic molecules have been reported to be delivered by CNTs recording better results than that obtained with conventional vehicles [42–44]. The high surface area of CNTs, indeed, allows us to achieve a high loading capacity of chemotherapy drugs [45]. Moreover, CNTs loaded with drugs can extravasate in tumor tissues over time because of the enhanced permeability and retention effect of solid tumors [46].

The remarkable importance of carbon nanotubes in biomedical field is related to their ability to undergo cell internalization. Nonetheless, the molecular mechanism of the interaction between CNTs and cells/tissues has to be explained.

Kam et al. [47, 48] propose that SWNTs traverse the cellular membrane via endocytosis, whereas Pantarotto et al. [49] suggest a nonendocytosis mechanism involving the insertion and diffusion of nanotubes through the lipid bilayer of the cell membrane. Several studies showed that high amounts of CNTs can be found in the cell matrices and in the nucleus after exposure to these nanomaterials [50]. This represents a starting point for the development of novel biomedical devices based on CNT nanostructure.

Regarding the drug delivery field, three different methods of interaction between CNTs and active compounds have been proposed: the first one is a porous absorbent to entrap active components within a CNT mesh or CNT bundle, the second approach is through functional attachment of the compound to the exterior walls of the CNTs, and the last one involves the use of CNT channels as nanocatheters.

Water solubility is an important prerequisite for gastrointestinal absorption, blood transportation, secretion, and biocompatibility; thus, CNT composites involved in therapeutic delivery system have to present this requirement. Similarly, it is important that such CNT dispersions should be uniform and stable in a sufficient degree, so as to obtain accurate concentration data. The solubilization of pristine CNTs in aqueous media is one of the key obstacles in the way for them to be developed as practical drug carriers owing to the rather hydrophobic character of the graphene side walls, coupled with the strong *π*-*π* interactions between the individual tubes. These properties cause aggregation of CNTs into bundles [51].

In order to achieve a successful dispersion of carbon nanotubes, the medium should be capable of both wetting and modifying the hydrophobic tube surfaces in the aim to decrease tube's bundle formation. Foldvari and Bagonluri have proposed four different strategies to obtain desirable dispersion [30]: (1) surfactant-assisted dispersion, (2) solvent dispersion, (3) functionalization of side walls, and (4) biomolecular dispersion. Among the above described approaches, functionalization has been the most effective approach. In addition, functionalization has been shown capable of decreasing cytotoxicity, improving biocompatibility, and giving opportunity to appendage molecules of drugs, proteins, or genes for the construction of delivery systems [40].

Furthermore, the CNT functionalization is of dramatic importance from a toxicological point of view.

As cited before, CNTs have the ability to be cytotoxic both in vivo and in vitro [52–54]. This is due to both the structural and physiochemical aspects of CNTs. As the particle size of a material decreases, indeed, there is a corresponding elevation in reactivity and hence toxicological and inflammatory potential [55]. This is because nanomaterials such as CNTs have relatively high surface area to volume ratios over which more chemical interactions can occur. Furthermore, nanomaterials share similar dimensional scales to many large biological molecules rendering them inherently more likely to disrupt natural interactions and cause toxicity [56, 57].

CNTs have demonstrated both positive [58] and negative [59] toxicological effects and that the mechanisms regulating their toxicity are still to be understood [60].

CNTs are believed to present the highest risk of adverse health effects to the respiratory system due to their light weight and propensity to be aerosolized and inhaled. In 2004, Lam et al. showed CNTs to cause granulomas in the lung tissue of mice as a result of intratracheal instillation [59]. Increasing doses corresponded to higher rates of inflammation and prominence of lesions. When compared to the quartz positive control, a known cause of granulomas and lung lesions, CNTs showed a higher incidence of negative health effects. This included increased inflammation, fibrosis, granulomas, necrosis, and lethality. Similar results have been demonstrated repeatedly [61]. Comparing more directly to asbestos, in which the mesothelium of the lungs is affected, Poland et al. exposed the mesothelial tissue lining the lung/body cavity of mice to CNTs with a median length of approximately 20 *μ*m [62]. Toxic behavior was evidenced including inflammation and the formation of granulomas.

Although a significant number of studies have been published regarding the activation of immune cells by CNTs, very few have focused on the immunogenicity of CNTs themselves [63]. Although it had been described for C60 fullerene derivatives [64], the induction of anti-CNT antibodies has not been evidenced so far, even when injected to mice in the presence of a strong immune adjuvant [65].

The biopersistence of CNTs warrants additional concerns when considering them for biological uses, particularly since this validates their comparison with the carcinogen asbestos. While this is concerning, the in vivo study of mice has demonstrated that “pristine” CNTs have a relatively low half-life of 3 hours in the bloodstream [66]. Through the use of various imaging techniques, the in vivo study of mice has also shown that CNTs pose risks specifically to the lungs, liver, and spleen persisting in these locations at up to 10% of the initial dose at the studies completion time of 28 days [67]. Furthermore, it is possible that the toxicological behavior of CNTs activates at a certain threshold meaning that this activation barrier would need to be identified and further dosage of CNTs limited according to the biopersistence [68]. Biopersistence depends on the functionalization. Carboxylated CNTs can be degraded not only in vitro using a model plant-derived enzyme, the horseradish peroxidase [69, 70], but also by the more physiologically relevant myeloperoxidase (MPO) present within granulocytes (mainly neutrophils) and to a lesser extent within macrophages [71]. Importantly, the MPO-degraded CNTs did not generate inflammation when aspirated into the lungs of mice [72].

## 3. CNTs-Hydrogel Hybrid Materials

As reported before, hydrogels have emerged as promising biomaterials due to their unique characteristics. These polymeric networks, indeed, resemble living tissues closely in their physical properties because of their relatively elastomeric and soft nature and high water content, minimizing mechanical and frictional irritation. These materials show a minimal tendency to cell adhesion and to absorption of proteins from body fluids due to their very low interfacial tension. In addition, the swelling capacity allows to easily remove reagent residues [73, 74].

Unfortunately, the same water content which makes hydrogels such a special class of materials is also responsible for their biggest disadvantage, the poor mechanical properties. Hydrogels with improved mechanical properties could be obtained through the preparation of interpenetrating polymer networks (IPN) by chemical cross-linking. However, the presence of residual cross-linking agents could lead to toxic side effects [75].

Burst or incomplete release of the therapeutic agent and the poor scalability of the manufacturing process represent other limitations in the use of hydrogel as drug delivery systems. In the aim to overcome these drawbacks, hydrogel composites materials have been developed [76]. The synthesis of these materials involves the incorporation of nanoparticles into a hydrogel matrix enhancing mechanical strength, drug release profile, remote actuation capabilities, and biological interactions [77]. The development of novel nanocomposite hydrogels was encouraged by the recent advances in the chemical, physical, and biological fields combined with rising needs in the biomedical and pharmaceutical sectors. Novel polymer chemistries and formulations as well as fabrication and processing techniques are supported by improved instrumentation that can measure and manipulate matter at the nanoscale level [78].

Hydrogel nanocomposites with different particulates, including clay, gold, silver, iron oxide, carbon nanotubes, hydroxyapatite, and tricalcium phosphate, have been synthesized and characterized to evaluate their potential application as biomaterials [79].

In 1994, the first polymer nanocomposites using carbon nanotubes as filler was reported [80]. In earlier nanocomposites, nanoscale fillers such as carbon blacks, silica, clays, and carbon nanofibers (CNF) were employed in the aim to enhance the mechanical, electrical, and thermal properties of polymers [81]. In the last years, carbon nanotubes have received much attention as suitable materials to enhance the electrical and mechanical properties of polymers [82].

The technology implications of using CNTs-hydrogel are significant to many fields, from semiconductor device manufacturing to emerging areas of nanobiotechnology, nanofluidics, and chemistry, where the ability to mold structures with molecular dimensions might open up new pathways to molecular recognition, drug discovery, catalysis, and molecule specific chemobiosensing [83].

Based on these considerations, the identification of the most effective synthetic strategy to prepare CNTs-hydrogel hybrid materials has been aroused by an increased and considerable interest.

The adopted synthetic approaches can be classified into covalent and noncovalent functionalization of carbon nanostructures with polymeric materials. The structure of CNTs in the final composite represents the main difference between these two strategies. In the noncovalent approach, no modification of the CNT structure occurs; thus, the properties of the whole composite are determined by the intrinsic properties of the starting CNT materials; in the covalent approach, a significant modification of the CNT surface is performed and it is responsible of the composite final properties.

The noncovalent functionalization approach is based on the molecular composition of CNTs. The sp^2^ bonded graphene structures at the sidewalls of CNTs, indeed, contain highly delocalized p electrons which can form functionalized CNTs with other p electron-rich compounds through *π*-*π* interaction. This organic functionalization method avoids modifying the intrinsic structures of CNTs and gives structurally intact CNTs with functionalities. Recently, the potential interaction between the highly delocalized *π*-electrons of CNTs and the *π*-electrons correlated with the lattice of the polymer skeleton has generated much interest and provided the motivation for studying the optical and electronic properties of composites of CNTs and *π*-conjugated polymers [84].

The main strategies for the synthesis of CNTs-polymer composites by covalent functionalization consist of “grafting to” and “grafting from” approaches [85, 86].

The first method involves the reaction of preformed polymeric chains with the surface of either pristine or prefunctionalized carbon nanotubes by performing radical or carbanion additions as well as cycloaddition reactions to the CNT double bonds. Since the curvature of the carbon nanostructures imparts a significant strain upon the sp^2^ hybridized carbon atoms that make up their framework, the energy barrier required to convert these atoms to sp^3^ hybridization is lower than that of the flat graphene sheets, making them susceptible to various addition reactions. Therefore, to exploit this chemistry, it is only necessary to produce a polymer centered transient in the presence of CNT material. Otherwise, the presence of defect sites on the surface of oxidized CNTs, as opened nanostructures with terminal carboxylic acid groups allow covalent linkages of oligomer or polymer chains. The “grafting to” approach onto CNT defect sites involves the reaction of the functional groups on the nanotube surfaces with the reactive end groups onto a readymade polymer. For this purpose, preformed commercial polymers of controlled molecular weight and polydispersity can be employed. The main limitation of this technique is that initial binding of polymer chains sterically hinders diffusion of additional macromolecules to the CNT surface, leading to a low grafting density.

The “grafting from” approach involves the polymerization of monomers from surface-derived initiators on CNTs. These initiators are covalently attached using the various functionalization reactions developed for small molecules, including acid-defect group chemistry and side-wall functionalization of CNTs. The polymer growth is not limited by steric hindrance; thus, this synthetic strategy allows us to obtain efficiently grafted polymers characterized by a high molecular weight. In addition, nanotube-polymer composites with quite high grafting density can be prepared. However, an accurate control of the amounts of initiator and substrate and of the polymerization reaction conditions is required by this methodology. Moreover, the continuous *π*-electronic properties of CNTs would be destructed by the acid oxidation, even worse; CNTs may be destroyed to several hundred nanometers in length. As a result, compared with the “grafting from,” the “grafting to” has much less alteration of the structure of CNTs [87, 88].

Many techniques including esterification [89], “click” chemistry [90], layer-by-layer self-assembly [91], pyrene moiety adsorption [92, 93], radical coupling [94, 95], anionic coupling [96], radical polymerization [97], supercritical CO_2_-solubilized polymerization or coating [98], **γ**-ray irradiation [99], cathodic electrochemical grafting [100] polycondensation [101, 102], reversible addition fragmentation chain-transfer (RAFT) polymerization [103, 104], anionic polymerization [105], ring-opening polymerization [106, 107], and atom transfer radical polymerization (ATRP) [108, 109] have been employed in the aim to functionalize CNTs with polymers.

### 3.1. CNTs-Hydrogels in Drug Delivery

All the above mentioned considerations clearly highlight the importance and, thus, the considerable interest of the scientific community to the use of composite materials based on carbon nanotubes and polymeric hydrogels to be applied as innovative drug delivery devices.

Nanohybrid hydrogels, indeed, combine properties of both hydrogels and CNTs, carrying out the preparation of devices with improved physic-chemical, mechanical, and biological properties [110].

For tissue regeneration, CNTs have been blended with both synthetic [111] and biological [112] tissue scaffolds in the aim to improve their mechanical properties and/or electrical conductance [113].

The ability of CNTs to lower the impedance of polymer matrices may be useful for the subset of tissues whereby electrical signals are propagated, namely, neural tissue and cardiac muscle. CNTs have been demonstrated to be able to improve neural signal transfer, while supporting dendrite elongation and cell adhesion [114] and the electrical conductivity of CNTs could be a useful tool for directing cell growth, as shown for the case of osteoblast proliferation in which electrical stimulation delivered through novel, current conducting polymer/nanophase composites promotes osteoblast functions that are responsible for the chemical composition of the organic and inorganic phases of bone [115]. Furthermore, application of electrical stimulation on the conducting matrix could be used to increase the rate of neurotrophin release, to augment nerve regeneration [112].

In addition to neural cells and osteoblasts, CNT composite matrices have also been shown to be useful scaffolds for the adhesion and the long-term growth of a variety of other cell types, ranging from skin fibroblasts [116] to muscle myoblasts [117].

CNTs were wrapped with various polysaccharides to systematically investigate the effect of hydrophilicity/hydrophobicity and surface chemistry on cell behavior. It was found that amylase-wrapped surfaces presenting OH groups were optimal to promote cell adhesion and viability [118]. CNTs covalently functionalized with phosphonate and sulfonate groups were able to nucleate hydroxyapatite (HA) and undergo mineralization, indicating their promise as a bone scaffold [119]. Differences in the surface chemistry and functionality of CNT substrates were also shown to affect osteoblast cell phenotype [120].

Among polysaccharides, chitosan is widely employed to prepare CNTs-hydrogels. A research study, indeed, was reported on the synthesis of chitosan hydrogel beads impregnated with carbon nanotubes (CNTs). Composite hydrogel beads were manufactured by dispersing CNTs (0.01 wt.%) with cetyltrimethylammonium bromide (0.05 wt.%) into chitosan solution (1 wt.%) and mechanical strength, acid stability and adsorption capacity to the anionic dye, Congo red, of the prepared materials were investigated. Maximum endurable force at complete breakdown of hydrogel beads increased from 1.87 to 7.62 N with incorporation of CNTs and its adsorption capacity increased from 178.32 to 423.34 mg g^−1^ for adsorption of Congo red [121].

Carbon nanotubes were also incorporated into gellan gum hydrogels as conducting fillers to achieve an electrically conducting hydrogel for electrical cell stimulation. The formation of hydrogels involves a conformational change from the disordered (random coil) to ordered (double helix) chain structure on cooling, followed by aggregation of helices to form a gel network in the presence of a sufficient crosslinker (cations) concentration. CNTs were incorporated by probe sonication and percolation studies revealed that a carbon nanotube concentration of 1.3% by weight is required to achieve electrical conduction through the hydrogel [122].

In another work, molecularly imprinted polymers (MIPs) suitable for the electroresponsive release of diclofenac sodium salt drug were synthesized by precipitation polymerization in the presence of carbon nanotubes (CNTs) [123], by a modified grafting approach [124]. Conventional and electroresponsive imprinted polymers were synthesized with methacrylic acid as the functional monomer and ethylene glycol dimethacrylate as the crosslinker. In vitro release experiments, performed in aqueous media, highlighted the ability of the MIPs and spherical imprinted polymers doped with CNTs to release the loaded template in a sustained manner over time in comparison to that of the corresponding nonimprinted materials.

In another study, CNTs were tested as reinforcing agent for polyvinyl alcohol (PVA) hydrogels, and the resulting composite material was found to elicit a stronger biological response than pure hydrogel in osteochondral defect repairing application. An increase in bone growth rate at the implant/tissue interface was detected, as a result of the increasing concentration of calcium and phosphorus in the implants over time, without any kind of significant inflammatory process after 12 weeks [125].

Specifically, the effect of MWNTs on the mechanical properties of hydrogels was also tested by preparing a composite material containing poly(vinyl alcohol) (PVA) and poly(vinyl pyrrolidone) (PVP) with wrapped multiwalled carbon nanotube (MWCNT). The results demonstrated that the mechanical properties of the composite hydrogel were improved significantly by adding MWCNTs. Particularly, a 133% improvement of the tensile strength and a 63% improvement of the tear strength were achieved by the addition of only 1.0 wt% of MWCNTs. In addition, all the MWCNT-PVP/PVA composite hydrogels became more wear-resistant in the presence of PVP with different MWCNT contents [126].

Different composite scaffolds composed of hydroxyapatite (HAp) and collagen were synthesized by an in situ precipitation method. The obtained scaffolds showed higher mechanical performance than pure collagen scaffold and hierarchical porosity and, thus, was regarded as a promising routine for bone regenerative application [127].

Inclusion of carbon nanotubes in polymeric hydrogels increases conductivity and decreases transition time. Specifically, the effect of CNTs on hydrogels prepared by simple free-radical copolymerization of commercially purchased monomers, such as sodium acrylate, sodium (4-styrene sulfonate), and polyethylene glycol diacrylate, physically crosslinked by means of metal ions, was explored. The used metal ions are the Fe^2+^/Fe^3+^ redox couple due to its ability to control the degree of cross-linking in a polymer bearing hard carboxylate side groups. Iron ions in different oxidation states, indeed, have distinct coordination preferences and Fe^3+^ binds more strongly than Fe^2+^ to “hard” ligands. Thus, the change in oxidation state can be used to modulate the cross-linking and the mechanical properties of the bulk material by the interconversion of Fe^2+^ and Fe^3+^. The conductivity of the hydrogel was improved by the addition of 1−3% vinyl-functionalized MWNTs and, as result, the charge versus time response changed dramatically. The time to pass 40 Coulombs decreased from 11.9 h for hydrogel with no nanotubes to 3.2 h for 3% MWNTs. Thus, the nanotubes improve conduction such that the distance that iron atoms must diffuse for reduction is decreased [128].

The same effect was recorded in nanotube-polymeric ionic liquids gels synthesized by noncovalent functionalization of oxidized single-walled carbon nanotube surfaces with imidazolium-based poly (ionic liquids) (PILs), using in situ radical polymerization method. The obtained hydrogel was characterized by high thermal stability, with the onset points of decomposition shifting to a higher temperature with increasing SWNT contents in the gel composites. By plotting the surface resistivity with respect to the loading content of SWNT in SWNT-PIL gel composites, a decrease in surface resistivity of gel composite (from 3.8 × 10^7^ to 1.7 × 10^3^ Ω/square) with an increase in the SWNT contents (from 0 to 1.5 wt.%) was recorded [129].

MWNTs were also found to significantly enhance the specific surface areas (280–400 m^2^/g), the thermal stability, and electrical conductivities (1.2–6.9·10^−2^ S/cm) of composite aerogels prepared by embedding carbon nanotubes into poly(3,4-ethylenedioxythiophene)—poly(styrenesulfonate) (PEDOT-PSS) supermolecular hydrogels in the presence of a very small amount of PVA. In this work, CNTs could be both in the pristine and in the oxidized state (by means of acidic treatment), while PVA is used to keep from aggregation of MWCNTs and hydrogels are synthesized by polymerizing 3,4-ethylenedioxythiophene with excessive ferric nitrate as oxidizing agent as well as cross-linking agent in the presence of poly(sodium 4-styrenesulfonate) [130].

Similarly, multiwalled carbon nanotubes were chemically modified with ethylene glycol to improve their dispersion in poly(3,4-ethylene dioxythiophene)-poly(4-styrenesulfonate). It was found that the functionalized CNTs, well dispersed in the composite material, formed a conductive network after spray-coating. The surface resistance and transmittance of the films decreased as the number of coatings increased. The surface resistance of the films was further decreased with the addition of polar solvents, while the current remarkably increased. It was noted that polar solvents having a high dielectric constant led to stabilization and high dispersity of the negative charged CNTs and provided a screening effect between the polymeric counterpart, resulting in an increase of the electric and electrochemical properties [131].

High-quality conductive composite hydrogels composed of SWNTs, polypyrrole (PPy), and poly(ethylene glycol) diacrylate (PEGDA) hydrogel were successfully prepared through interfacial polymerization (IP). Compared to the conventional sequential interpenetrating polymerization (CI), IP is able to better improve the electrical/electrochemical properties of the incorporated hydrogel due to the higher content of PPy up to 14.1 wt% in its dry weight. The electrical conductivity of PPy/PEGDA hydrogel is nearly more than two orders of magnitude higher than that of the hydrogel prepared via CI. In particular, the electron-transfer resistance (Rct) dramatically decreased from 9320 U for the pure hydrogel to 247 U for the SWNT/PPy/PEGDA hydrogel. It was also found that the addition of SWNTs and PPy would not noticeably decrease the swelling ratio, while significantly improving the mechanical and electrical properties of the pure PEGDA hydrogel. The composite hydrogel effectively integrates the remarkable electrical/electrochemical properties from SWNTs and PPy with excellent biocompatibility from the hydrogel [132].

The importance in retaining of the typical swelling properties of hydrogels was highlighted in a work in which single-walled carbon nanotubes were incorporated into a poly(ethylene glycol) (PEG) hydrogel by covalent bonds. In particular, SWNTs were functionalized with poly(ethylene glycol) methacrylate (PEGMA) to obtain water-soluble pegylated SWNTs (SWNT-PEGMA) and then the functionalized SWNTs were covalently bonded through their PEG moieties to a PEG hydrogel. The hybrid network was obtained from the cross-linking reaction of poly(ethylene glycol) diacrylate prepolymer and SWNT-PEGMA by dual photo-UV and thermal initiations and their swelling properties were found to be maintained when compared with the native PEG hydrogel [133].

In another research study, the influence of CNTs on swelling behavior and wettability was studied by preparing a hybrid structure of aligned carbon nanotubes and a pH-responsive hydrogel. The composite was prepared by performing a chemical vapor deposition of a P(MAA-co-EGDA) hydrogel directly on carbon nanotube arrays with low site density from monomer vapors. The process allowed preservation of the monomer functionality, retention of the nanotube alignment, and control of the coating thickness at the sub-100 nm level. The P(MAA-co-EGDA) hydrogel demonstrated pH-responsive swelling on both planar surfaces and carbon nanotube arrays and no appreciable swelling was recorded when immersing the hydrogel in acidic solutions. A swelling ratio of 38%, defined as the thickness increase relative to the dry thickness, was observed at pH 7 indicating the ionization of carboxyl groups in the P(MAA-co-EGDA) hydrogel. At the same time, the coated nanotube arrays showed a smoother surface due to the expansion of the hydrogel. The hydrogel coating significantly enhanced the wettability of the CNT surface. The wettability of the coated VACNTs depended on both the coating thickness and the pH conditions. At pH 2, the wettability of the coated CNTs with different coating thicknesses was in agreement with the calculated results of the Cassie-Baxter model. Under neutral pH conditions, the coated VACNT surface exhibited an apparent contact angle of nearly zero. The superwettability was attributed to the synergistic effect of the structure porosity and the ionization of the pH-responsive hydrogel [134].

The influence of CNTs on cyclodextrins-based hydrogel was also matter of studies in several articles.

Chemically responsive supramolecular SWNT hydrogel was prepared by using soluble SWNTs functionalized cyclodextrin moieties on SWNT surface. Since cyclodextrin shows high solubility in water, water-soluble SWNTs carrying cyclodextrin are obtained by using *π*-*π* interaction between pyrene modified cyclodextrin and SWNTs. Cyclodextrin forms host guest complexes with various kinds of guest compounds; thus, vacant cavities in the composite are able to capture guest molecules on SWNT surface [135].

In another work, poly(ethylene glycol)-grafted-multiwalled carbon nanotube (MWNT-g-PEG) was synthesized by a coupling reaction and formed inclusion complexes after selective threading of the PEG segment of the MWNT-g-PEG through the cavities of *α*-cyclodextrins units. The complexation of the PEG segments with cyclodextrin and the hydrophobic interaction between the MWNTs resulted in the formation of supramolecular hybrid hydrogels with a strong network. Thermal analysis showed that the thermal stability of the hydrogel was substantially improved by up to 100°C higher than that of native hydrogel. The mechanical strength of the hybrid hydrogels was also greatly improved in comparison with that of the corresponding native hydrogels. The resultant hybrid hydrogels were found to be thixotropic and reversible and could be applied as a promising injectable drug delivery system [136].

CNT was hybridized into a supramolecular hydrogel based on the selective inclusion of *α*-cyclodextrin onto poly(ethylene oxide) (PEO) segments of a triblock copolymer, that is, PEOblock-poly(propylene oxide)-block-PEO. The network density of hydrogel was designedly reduced, decreasing the content of *α*-cyclodextrin, in order to improve the diffusion of encapsulated substances, while CNT played a key role in mechanical reinforcement resulting in the increase of storage modulus. In addition, the incorporation of CNT could decrease the gelation time, and the CNT hybridized hydrogel still kept the shear-thinning property. Cell viability assays confirmed the equivalent cytotoxicity of the supramolecular hybrid hydrogels to that of the native hydrogels without CNT. Consequently, the synthesized CNT-hybridized supramolecular hydrogel shows a great potential for biomedical applications [137].

In the literature, several studies aim to evaluate the effect of introducing CNTs into polyacrylamide-based hydrogels. Different hydrogels synthesized by using acrylamide as monomer and methylenebisacrylamide and/or tetramethylethylenediamine as crosslinkers were prepared by free radical cross-linking copolymerization in water [138, 139]. Similarly, the corresponding composite materials were prepared by introducing MWNTs into the polymerization feed at different amount (%), ranging from 0.1% to 15% and 50% [83].

It was observed that high MWNT content composites dry much faster, as the result of having larger desorption coefficients for all measurements, while a slower swelling is recorded because of a smaller diffusion coefficients of solvent in the polymeric network [138].

The mechanical properties of MWNT-polymer composites were found to be highly dependent on nanotube dispersion, which directly influences the molecular tube-tube and tube-polymer interactions in the composites. Such molecular interactions will play a critical role in load transfer and interfacial bonding that determines mechanical properties of the materials. The variations in the nanotube dispersion in the resultant composite could be the major reason for this phenomenon, while the increasing MWNT content produces infinite network, reducing the swelling and decreasing compressive elastic modulus [139].

Regarding the electrical properties, the addition of carbon nanotubes to polymer systems with an isolator character leads to the formation of electrically conducting composite structures. For this purpose, CNT addition has to exceed a critical value [83].

The effect of CNT addition on thermally responsive hydrogels was shown in the following reported studies [140, 141].

Reversible, thermally and optically responsive actuators were prepared utilizing composites of poly(N-isopropylacrylamide) (pNIPAM) loaded with single-walled carbon nanotubes. Uniform SWNT dispersion in the p-NIPAM hydrogels was achieved by means of sodium deoxycholate solution as surfactant. With nanotube loading at concentrations of 0.75 mg/mL, authors demonstrated up to 5 times enhancement to the thermal response time of the nanotube-pNIPAM hydrogel actuators caused by the enhanced mass transport of water molecules. Additionally, the ability to obtain ultrafast near-infrared optical response in nanotube-pNIPAM hydrogels under laser excitation enabled by the strong absorption properties of nanotubes was shown [140].

A nanocomposite of multiwalled carbon nanotubes and temperature responsive N-isopropylacrylamide hydrogels was prepared by using Tetra (ethylene glycol) dimethacrylate as crosslinker in a radical polymerization method. Various amounts of acrylamide (AAm) were added to modulate the lower critical solution temperature (LCST) of the nanocomposites for tailored physiological applications. An increase in the amount of AAm shifted the LCST transition to higher temperatures, while the addition of nanotubes contributed to interesting properties, including tailorability of temperature responsive swelling and mechanical strength of the resultant nanocomposites. The addition of MWCNTs significantly decreased the swelling ratios, due to the hydrophobic behaviour, while it did not affect the LCST transition temperature range [77].

Hybrid composite hydrogels were also synthesized by using gelatin as starting materials and the effect of CNTs on the swelling properties of the resultant materials was evaluated. The choice of gelatin is of great importance by virtue of its advantageous physical, chemical, and biological properties, which allow us a wide range of applications in food science, biomedical, and pharmaceutical field [141–143].

In particular, a hybrid gelatin hydrogel with carbon nanotubes was synthesized by physical mixing method and studied in terms of water affinity behavior. Within the first 5 min, the swelling ratio of hybrid gel was higher than that of native gelatin gel due to the capillarity of MWNTs in the matrix, which is help for the solvent diffusing into the gel matrix. After this time interval, the hybrid gel was slower to reach swelling equilibrium than that of pure gelatin gel. This indicated that MWNTs are able to inhibit the swelling of gel matrix by inhibiting the dissolution of gelatin in the matrix. This effect of MWNTs can be called a shielding effect and it represents the leading effect after the first 5 min [35].

The increase in the electroconductivity of gelatin containing hybrid hydrogels was also proved and multiwalled carbon nanotube (MWNTs)/gelatin composites were prepared by dispersion of MWNTs through ultrasonication in an aqueous medium in the presence of sodium dodecyl sulfate as anionic surfactant. The response of the composite and pure hydrogel to the applied electrical field was investigated. Both the composite and pure hydrogel showed a two-stage bending phenomenon, an early bending towards the anode and a later bending towards the cathode. The incorporation of MWNTs gradually decreased the swelling of the hydrogel and exerted no effect on the swelling mechanism, which followed the second order kinetic. The bending mechanism can be explained on the osmotic pressure difference at the solution gel interface. Bending towards the anode at the first stage is attributed to the accumulation of the positive ions at the anode side of the gel film in the solution. The accumulated ions created osmotic pressure difference at the anode side. The osmotic pressure difference causes more water to penetrate into gel at the cathode side than the anode side leading the gel to bend towards the anode. The later bending towards the cathode may be attributed to the diffusion of the positive ions into the gel which creates an osmotic pressure difference on the cathode side of the gel. The composite showed good reversible behavior compared to the gelatin which might be due to the condensed structure of the composite compared to the gelatin, which erodes to a great extent on exposure to DC current for longer time. Both the bound and unbound water contents (determined by differential scanning calorimetry) decreased with the addition of MWNTs. This phenomenon might be due to the hydrophobic effect of the MWNTs and increase in cross-linking density between the MWNTs and gelatin by increasing the MWNT concentration [144].

**Figure 2:**
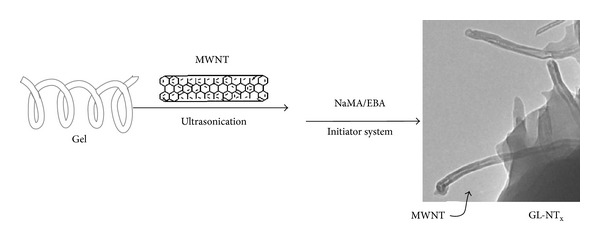
Synthesis of electroresponsive gelatin hybrid microgels.

In a different work, authors designed synthesized and characterized electroresponsive composite microgels to be inserted into a suitable topical drug delivery device in order to release diclofenac sodium salt therapeutics as consequence of an applied external voltage [145]. The hybrid hydrogels, composed of gelatin and multiwalled carbon nanotubes were synthesized by a modified grafting approach and employing an emulsion polymerization method in the presence of sodium methacrylate and N,*N*′-ethylenebisacrylamide. Different amounts of nanotubes (up to 35% by weight) were covalently inserted into the polymeric network in order to determine the percentage conferring the highest electric sensitivity to the composite microspheres (Figure 2). The swelling properties of microgels confirm the polyelectrolyte behavior of hydrogels, and the application of an external electric field (at pH 7.4) caused a reduction of the swelling degree from 1350 to 420% as a consequence of a built-in osmotic pressure. Drug release experiments demonstrated the ability of the most responsive composite to control diclofenac sodium salt release over time. The electric stimulation resulted in a further increase of the release (+20%) in MWNTs containing materials.

Similarly to the gelatin hydrogels, Fmoc-protected amino acid based hydrogel has been used to incorporate and disperse functionalized single-walled carbon nanotube within the gel phase to make a hybrid hydrogel a more stable, elastic, and conductive material than the native gel [146].

An electro-responsive transdermal drug delivery system was prepared by electrospinning of poly(vinyl alcohol)/poly(acrylic acid)/multiwalled carbon nanotubes (MWCNTs) nanocomposites. The surface modification of MWCNTs was carried out by oxyfluorination to introduce the functional groups on the hydrophobic MWCNTs. The electrical conductivity increased significantly by incorporating the oxyfluorinated MWCNTs and the more MWCNT content gave the higher electrical conductivity. The nanofiber showed the higher electrical conductivity when MWCNTs were oxyfluorinated with higher oxygen content due to the improved dispersion of MWCNTs in the polymer matrices. PVA/PAA/MWCNT nanofibers showed more than 80% cell viability and were tested as drug delivery device. The drug release behavior of nanofibers showed the similar trend with that of swelling behavior. The amount of released drug, which was related to the swelling of the system, increased by increasing the content of MWCNTs and oxyfluorination with higher oxygen content and by applying an external electric field [147].

The swelling ratio of nanofibers decreased with increasing the MWCNT content due to the restriction of swelling in the presence of MWNTs. The additional physical cross-linking resulting from the favorable interaction between MWCNTs and hydrophilic polymers contributed partly to the reduction of swelling of nanofibers. A totally different swelling behavior occurred by applying electric field on the hydrogel nanofibers. The swelling ratio of nanofibers increased under electric field with increasing the MWCNT content; thus, MWCNTs could make the efficient pathway of electric field due to their own electrical conductivity. This role of MWCNTs contributed a large share to increasing the swelling of nanofibers by accelerating the ionization of functional groups in the hydrophilic polymers.

In a similar work, authors explored the dual-stimuli sensitivity of MWCNT/PVA/PAAc composite microcapsules prepared by using multiwalled carbon nanotubes (MWCNTs), poly(vinyl alcohol) (PVA), and poly(acrylic acid) (PAAc). PAAc was extensively studied as a pH-sensitive polymer and its pendant carboxylic acid groups are generally ionized into carboxylate anions above its pKa of 4.7. The carboxylate anions cause more electrostatic repulsion and hydrophilicity to the polymer segments in the hydrogel. Therefore, as the pH increased, the PVA/PAAc hydrogels swelled more rapidly due to a large swelling driving force caused by the electrostatic repulsion between the ionized carboxylate groups. The release amount of an anionic model drug, such as coomassie brilliant blue (CBB), increased in a basic buffer solution due to the higher swelling of microcapsule shell. The drug release behavior was closely related to the morphology of composite microcapsules. When the MWCNTs were dispersed uniformly in the composite microcapsule, the drug was released more effectively under the electric field applied and this was attributed to the electrical networks of MWCNTs in the composite microcapsules, which were effective to respond to the electric field. The increased conductivity of composite microcapsule by MWCNTs could accelerate the ionization of carboxyl groups in polymer chains. The increased degree of ionization of polyelectrolyte hydrogel under electric field was reported to be responsible for the higher swelling ratio due to the more electrostatic repulsion and hydrophilicity in the polymer chains [27].

Carboxymethyl guar gum (CMG) chemically modified MWNT hybrid hydrogels were synthesized at different MWNT levels as potential device for sustained transdermal release of diclofenac sodium. The hydrogel was synthesized by noncovalent functionalization of acid-treated CNT with carboxymethyl guar gum. Spectroscopy together with morphology, thermogravimetry, and rheological studies proved relatively strong CMG-MWNT interaction at 0.5 and 1 wt% levels of MWNT whereas de-wetting was increased with higher MWNT concentration. Drug encapsulation tendency increased with addition of MWNT; maximum entrapment was noticed at 1 wt% MWNT level, while at higher MWNT level, the interaction level falls especially at high temperature. Presence of MWNTs enhances diclofenac sodium retention efficiency inside the hydrogel; maxima been shown at 1 wt% level. Hydrogels containing 0.5, 1, and 3 wt% MWNT exhibited slower transdermal release than neat CMG due to slightly higher gel viscosity and more drug entrapment. Slowest but steady release following non-Fickian type mechanism was obtained from 1 wt% MWNT loaded hydrogel due to highest viscous resistance among all other hybrid nanocomposites [8].

**Figure 3:**
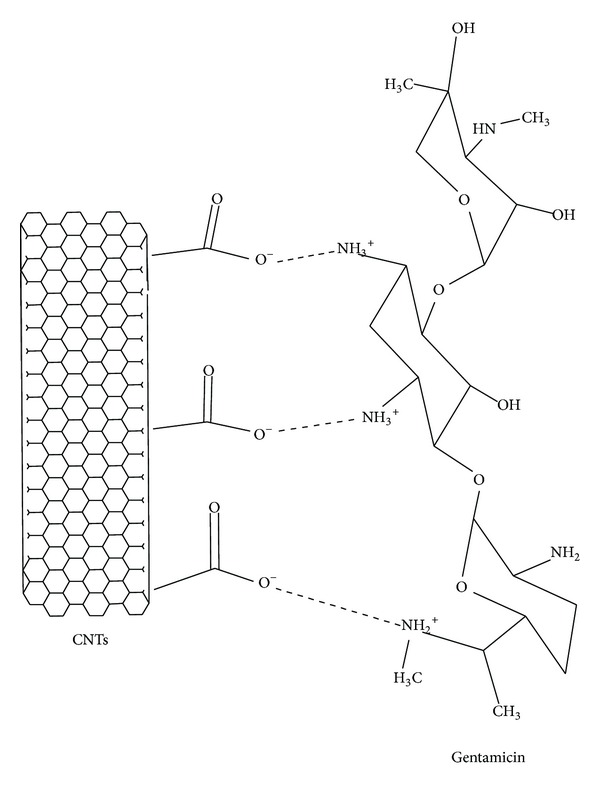
Interactions of CNTs with gentamicin sulphate.

The sustained release effects of a hybrid hydrogel on gentamicin sulphate were significantly improved. The release kinetics was characterized by an initial period of rapid release during the first 1 day followed by a nearly constant slow release rate. It can be assumed that initially the unbound portion of gentamicin sulphate in the matrix diffused through the hydrogel. After this stage, desorption of matrix-bound gentamicin sulphate becomes the limiting route for the drug transport across the hydrogel. The better release effect is obtained by the CNTs hybrid hydrogel, due to the many sites of CNTs suitable for drug adsorption (Figure 3) [34].

An electrosensitive transdermal drug delivery system was also prepared by the electrospinning method to control drug release. A semi-interpenetrating polymer network was prepared as the matrix with polyethylene oxide (PEO) and pentaerythritol triacrylate polymers (PETA). Multiwalled carbon nanotubes were used as an additive to increase the electrical sensitivity and the release experiment was carried out under different electric voltage conditions. Drug release was effectively increased with increasing applied electric voltage, due to the excellent conductivity of the carbon additives. Water insoluble crosslinked PETA polymers form the main structure of the composite device, and water-soluble PEO is within the network of PETA polymers. The drugs are trapped among the copolymers and the resultant semi-IPN structure allows for the retarded solvation of the PEO polymer due to the physical trapping effect of PEO in the PETA network. The main mechanism of drug release from the copolymer network is by dissolving the PEO polymers. Dissolution of PEO polymers, indeed, creates empty spaces, which form the route for releasing the drug. The polymer weight loss was assessed and was found to increase significantly with increasing applied electric voltage. This was related to the fact that solvation of PEO might be accelerated due to the more rapid movement of ions and the swelled PETA polymers when high electric voltage is applied to the whole system [148].

The preparation of an electroresponsive multiwalled carbon nanotube/poly(methylacrylic acid) (MWNT/PMAA)-based hybrid material is reported in a recent work [149]. The importance of this study is that, for the first time, it was possible to carry out in vivo experiments to prove the possibility to achieve a controlled drug release upon the ON/OFF application of an electric field, giving pulsatile release profiles.

Particular kinds of hybrid hydrogels are those based on amyloid fibrils [150] and DNA [151].

**Figure 4:**
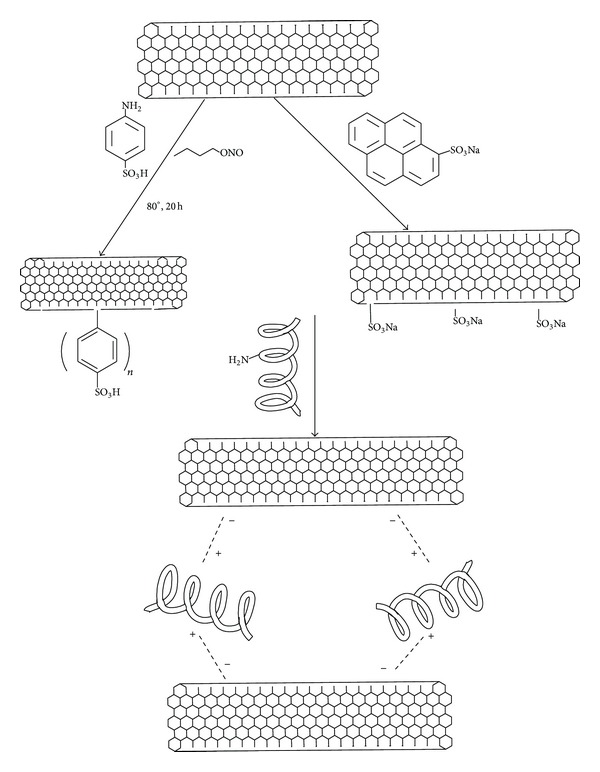
Schematic illustrations of functionalization of carbon nanotubes by amyloid fibrils.

Biocompatible, pH-responsive, and fully fibrous hydrogels have been prepared based on amyloid fibrils hybridized and gelled by functionalized MWNTs far below the gelling concentration of amyloid fibrils. The sulfonic functional groups were introduced on the surfaces of MWNTs either by a covalent pathway, using a mild and environmental friendly diazonium reaction or by a physical procedure via pyrene sulfonic acid capable of binding MWNTs via *π*-*π* interactions (Figure 4). The resulting hydrogels exhibit reversible pH-response, gelling at acidic pH lower that the pI of the amyloid fibrils, and flowing at pH > pI. Individual MWNTs can bind several amyloid fibrils at different positions, leading to multiple physical interaction points. This clearly indicates that MWNTs can act as efficient cross-linkers in a dispersion of amyloid fibrils. Indeed, at higher concentrations and at pH 2, the amyloid fibrils did complex with MWNTs, leading to an interconnected network in water. Since many proteins can form amyloid fibrils with various properties, this study provides the potential to prepare responsive hydrogels on demand, which could serve in biological applications, drug release, sensors, and tissue engineering [150].

DNA hydrogels, formed entirely by unconstrained branched DNA nanostructures, are emerging as promising materials for biomedical application. DNA has been, indeed, proved as ideal molecules in constructing precisely controllable two- and three-dimensional DNA nanostructures. In a recent work, authors report on the preparation of a DNA-single walled carbon nanotube (SWNT) hybrid hydrogel, which is pH-responsive and strength tunable. The DNA-SWNT hybrid hydrogel consists of two components: the linear unit is a 12 bp long duplex with a sticky domain at each end and the cross-linking unit is SWNT wrapped by specially designed DNA structures which can leave multiple sticky domains sticking out. This hydrogel is able to change into sol state within one minute at pH 8.0 [151].

Finally, the possibility to obtain interesting hydrogels by using CNTs alone and nanotube-graphene hybrid hydrogels should be cited.

Hydrogels based on CNTs were prepared by oxidizing ingle-walled carbon nanotubes by a technique previously developed for the oxidation of graphite to graphite oxide. This process involves treatment with concentrated H_2_SO_4_ containing (NH_4_)_2_S_2_O_8_ and P_2_O_5_, followed by H_2_SO_4_ and KMnO_4_. Oxidation results in complete exfoliation of nanotube ropes to yield individual oxidized tubes that are 40–500 nm long. The oxidized nanotubes slowly form viscous hydrogels at unusually low concentration (0.3 wt %), and this behavior is attributed to the formation of a hydrogen-bonded nanotube network. The oxidized tubes bind readily to amine-coated surfaces, on which they adsorb as smooth and dense monolayer films. Thin films of the oxidized nanotubes show ohmic current-voltage behavior, with resistivities in the range of 0.2–0.5 Ohm-cm [152].

Another research study focused on the preparation of carbon nanotube-graphene hybrid aerogels by supercritical CO_2_ drying of the hydrogel precursors obtained from heating the aqueous mixtures of graphene oxide and carbon nanotubes with vitamin C without stirring. The resulting hybrid aerogels showed very promising performance in water purification including capacitive deionization of light metal salts, removal of organic dyes, and enrichment of heavy metal ions. Assembling graphene and CNT hybrid aerogels, which may integrate the intriguing properties of graphene sheets and CNTs and unique characteristics of aerogels, would create new bulk materials with desired structures and charming properties [153].

## References

[1] Hughes GA (2005). Nanostructure-mediated drug delivery. *Nanomedicine: Nanotechnology, Biology, and Medicine*.

[2] Cirillo G, Iemma F, Puoci F (2009). Imprinted hydrophilic nanospheres as drug delivery systems for 5-fluorouracil sustained release. *Journal of Drug Targeting*.

[3] Satarkar NS, Biswal D, Hilt JZ (2010). Hydrogel nanocomposites: a review of applications as remote controlled biomaterials. *Soft Matter*.

[4] Morrow KJ, Bawa R, Wei C (2007). Recent advances in basic and clinical nanomedicine. *Medical Clinics of North America*.

[5] Wan ACA, Ying JY (2010). Nanomaterials for in situ cell delivery and tissue regeneration. *Advanced Drug Delivery Reviews*.

[6] Yang L, Zhang X, Ye M (2011). Aptamer-conjugated nanomaterials and their applications. *Advanced Drug Delivery Reviews*.

[7] Daum N, Tscheka C, Neumeyer A, Schneider M (2012). Novel approaches for drug delivery systems in nanomedicine: effects of particle design and shape. *Wiley Interdisciplinary Reviews: Nanomedicine and Nanobiotechnology*.

[8] Giri A, Bhowmick M, Pal S, Bandyopadhyay A (2011). Polymer hydrogel from carboxymethyl guar gum and carbon nanotube for sustained trans-dermal release of diclofenac sodium. *International Journal of Biological Macromolecules*.

[9] Singh B, Pal L (2008). Development of sterculia gum based wound dressings for use in drug delivery. *European Polymer Journal*.

[10] Sorbara L, Jones L, Williams-Lyn D (2009). Contact lens induced papillary conjunctivitis with silicone hydrogel lenses. *Contact Lens and Anterior Eye*.

[11] Wu J, Wei W, Wang L-Y, Su Z-G, Ma G-H (2007). A thermosensitive hydrogel based on quaternized chitosan and poly(ethylene glycol) for nasal drug delivery system. *Biomaterials*.

[12] Samchenko Y, Ulberg Z, Korotych O (2011). Multipurpose smart hydrogel systems. *Advances in Colloid and Interface Science*.

[13] Kost J, Langer R (2001). Responsive polymeric delivery systems. *Advanced Drug Delivery Reviews*.

[14] Zhang L, Zhao J, Zhu J, He C, Wang H (2012). Anisotropic tough poly(vinyl alcohol) hydrogels. *Soft Matter*.

[15] He H, Li L, Lee LJ (2008). Photopolymerization and structure formation of methacrylic acid based hydrogels: the effect of light intensity. *Reactive and Functional Polymers*.

[16] Zhu A, Shi Z, Jin J, Li G, Jiang J (2012). Synthesis and properties of polyacrylamide-based conducting gels with enhanced mechanical strength. *Journal of Macromolecular Science B*.

[17] Samanta S, Das S, Layek R, Chatterjee D, Nandi A (2012). Polythiophene-g-poly(dimethylaminoethyl methacrylate) doped methyl cellulose hydrogel behaving like a polymeric AND logic gate. *Soft Matter*.

[18] Li P, Dou X, Tang Y (2012). Gelator-polysaccharide hybrid hydrogel for selective and controllable dye release. *Journal of Colloid and Interface Science*.

[19] Della Rocca D, Willenberg B, Ferreira L (2012). A degradable, bioactive, gelatinized alginate hydrogel to improve stem cell/growth factor delivery and facilitate healing after myocardial infarction. *Medical Hypotheses*.

[20] Bobokalonov DT, Mukhidinov ZK, Rakhimov IF, Khodzhaeva FM, Kasymova GF, Liu LS (2012). Piroxicam ex vivo release kinetics from zein/pectin delivery systems. *Pharmaceutical Chemistry Journal*.

[21] Bernkop-Schnürch A, Dünnhaupt S (2012). Chitosan-based drug delivery systems. *European Journal of Pharmaceutics and Biopharmaceutics*.

[22] Zhang L-M, Wu C-X, Huang J-Y, Peng X-H, Chen P, Tang S-Q (2012). Synthesis and characterization of a degradable composite agarose/HA hydrogel. *Carbohydrate Polymers*.

[23] Hoffman AS (2002). Hydrogels for biomedical applications. *Advanced Drug Delivery Reviews*.

[24] Kim SJ, Yoon SG, Lee YM, Kim SI (2003). Electrical sensitive behavior of poly(vinyl alcohol)/poly (diallyldimethylammonium chloride) IPN hydrogel. *Sensors and Actuators B*.

[25] Li H, Yuan Z, Lam KY (2004). Model development and numerical simulation of electric-stimulus-responsive hydrogels subject to an externally applied electric field. *Biosensors and Bioelectronics*.

[26] Meng Q, Hu J (2009). A review of shape memory polymer composites and blends. *Composites Part A*.

[27] Yun J, Im JS, Lee Y-S, Bae T-S, Lim Y-M, Kim H-I (2010). PH and electro-responsive release behavior of MWCNT/PVA/PAAc composite microcapsules. *Colloids and Surfaces A*.

[28] Zhang W, Zhang Z, Zhang Y (2011). The application of carbon nanotubes in target drug delivery systems for cancer therapies. *Nanoscale Research Letters*.

[29] Cirillo G, Hampel S, Klingeler R (2011). Antioxidant multi-walled carbon nanotubes by free radical grafting of gallic acid: new materials for biomedical applications. *Journal of Pharmacy and Pharmacology*.

[30] Foldvari M, Bagonluri M (2008). Carbon nanotubes as functional excipients for nanomedicines: I. pharmaceutical properties. *Nanomedicine: Nanotechnology, Biology, and Medicine*.

[31] Donaldson K, Aitken R, Tran L (2006). Carbon nanotubes: a review of their properties in relation to pulmonary toxicology and workplace safety. *Toxicological Sciences*.

[32] Peigney A, Laurent C, Flahaut E, Bacsa RR, Rousset A (2001). Specific surface area of carbon nanotubes and bundles of carbon nanotubes. *Carbon*.

[33] Taylor A, Lipert K, Krämer K (2009). Biocompatibility of iron filled carbon nanotubes in vitro. *Journal of Nanoscience and Nanotechnology*.

[34] Li H, He J, Zhao Y, Wang G, Wei Q (2011). The effect of carbon nanotubes added into bullfrog collagen hydrogel on gentamicin sulphate release: in vitro. *Journal of Inorganic and Organometallic Polymers and Materials*.

[35] Li H, Wang DQ, Liu BL, Gao LZ (2004). Synthesis of a novel gelatin-carbon nanotubes hybrid hydrogel. *Colloids and Surfaces B*.

[36] Erlanger BF, Chen B-X, Zhu M, Brus L (2001). Binding of an anti-fullerene igg monoclonal antibody to single wall carbon nanotubes. *Nano Letters*.

[37] Balavoine F, Schultz P, Richard C, Mallouh V, Ebbesen TW, Mioskowski C (1999). Helical crystallization of proteins on carbon nanotubes: a first step towards the development of new biosensors. *Angewandte Chemie*.

[38] Mattson MP, Haddon RC, Rao AM (2000). Molecular functionalization of carbon nanotubes and use as substrates for neuronal growth. *Journal of Molecular Neuroscience*.

[39] Guo Z, Sadler PJ, Tsang SC (1998). Immobilization and visualization of DNA and proteins on carbon nanotubes. *Advanced Materials*.

[40] Foldvari M, Bagonluri M (2008). Carbon nanotubes as functional excipients for nanomedicines: II. Drug delivery and biocompatibility issues. *Nanomedicine: Nanotechnology, Biology, and Medicine*.

[41] Zhang Y, Bai Y, Yan B (2010). Functionalized carbon nanotubes for potential medicinal applications. *Drug Discovery Today*.

[42] Klumpp C, Kostarelos K, Prato M, Bianco A (2006). Functionalized carbon nanotubes as emerging nanovectors for the delivery of therapeutics. *Biochimica et Biophysica Acta*.

[43] Bianco A, Kostarelos K, Prato M (2005). Applications of carbon nanotubes in drug delivery. *Current Opinion in Chemical Biology*.

[44] Pastorin G (2009). Crucial functionalizations of carbon nanotubes for improved drug delivery: a valuable option?. *Pharmaceutical Research*.

[45] Liu Z, Sun X, Nakayama-Ratchford N, Dai H (2007). Supramolecular chemistry on water- Soluble carbon nanotubes for drug loading and delivery. *ACS Nano*.

[46] Maeda H, Bharate GY, Daruwalla J (2009). Polymeric drugs for efficient tumor-targeted drug delivery based on EPR-effect. *European Journal of Pharmaceutics and Biopharmaceutics*.

[47] Kam NWS, Dai H (2005). Carbon nanotubes as intracellular protein transporters: generality and biological functionality. *Journal of the American Chemical Society*.

[48] Kam NWS, Liu Z, Dai H (2006). Carbon nanotubes as intracellular transporters for proteins and DNA: an investigation of the uptake mechanism and pathway. *Angewandte Chemie*.

[49] Pantarotto D, Partidos CD, Graff R (2003). Synthesis, structural characterization, and immunological properties of carbon nanotubes functionalized with peptides. *Journal of the American Chemical Society*.

[50] Porter AE, Gass M, Muller K, Skepper JN, Midgley PA, Welland M (2007). Direct imaging of single-walled carbon nanotubes in cells. *Nature Nanotechnology*.

[51] Bianco A, Kostarelos K, Prato M (2011). Making carbon nanotubes biocompatible and biodegradable. *Chemical Communications*.

[52] Gilmour AD, Green RA, Thomson CE (2013). A low-maintenance, primary cell culture model for the assessment of carbon nanotube toxicity. *Toxicological & Environmental Chemistry*.

[53] Helland A, Wick P, Koehler A, Schmid K, Som C (2007). Reviewing the environmental and human health knowledge base of carbon nanotubes. *Environmental Health Perspectives*.

[54] Snyder-Talkington BN, Qian Y, Castranova V, Guo NL (2012). New perspectives in vitro risk assessment of multiwalled carbon nanotubes: application of coculture and bioinformatics. *Journal of Toxicology and Environmental Health B*.

[55] Oberdörster G, Oberdörster E, Oberdörster J (2005). Nanotoxicology: an emerging discipline evolving from studies of ultrafine particles. *Environmental Health Perspectives*.

[56] Shi X, Sitharaman B, Pham QP (2008). In vitro cytotoxicity of single-walled carbon nanotube/biodegradable polymer nanocomposites. *Journal of Biomedical Materials Research A*.

[57] Newman P, Minett A, Ellis-Behnke R, Zreiqat H (2013). Carbon nanotubes: their potential and pitfalls for bone tissue regeneration and engineering. *Nanomedicine*.

[58] Sitharaman B, Shi X, Walboomers XF (2008). In vivo biocompatibility of ultra-short single-walled carbon nanotube/biodegradable polymer nanocomposites for bone tissue engineering. *Bone*.

[59] Lam C-W, James JT, McCluskey R, Hunter RL (2004). Pulmonary toxicity of single-wall carbon nanotubes in mice 7 and 90 days after intractracheal instillation. *Toxicological Sciences*.

[60] Kang S, Herzberg M, Rodrigues DF, Elimelech M (2008). Antibacterial effects of carbon nanotubes: size does matter!. *Langmuir*.

[61] Warheit DB (2006). What is currently known about the health risks related to carbon nanotube exposures?. *Carbon*.

[62] Poland CA, Duffin R, Kinloch I (2008). Carbon nanotubes introduced into the abdominal cavity of mice show asbestos-like pathogenicity in a pilot study. *Nature Nanotechnology*.

[63] Dumortier H (2013). When carbon nanotubes encounter the immune system: desirable and undesirable effects. *Advanced Drug Delivery Reviews*.

[64] Byrne MT, Guin’Ko YK (2010). Recent advances in research on carbon nanotube—polymer composites. *Advanced Materials*.

[65] Pantarotto D, Partidos CD, Hoebeke J (2003). Immunization with peptide-functionalized carbon nanotubes enhances virus-specific neutralizing antibody responses. *Chemistry and Biology*.

[66] Singh R, Pantarotto D, Lacerda L (2006). Tissue biodistribution and blood clearance rates of intravenously administered carbon nanotube radiotracers. *Proceedings of the National Academy of Sciences of the United States of America*.

[67] Yang K, Wan J, Zhang S, Zhang Y, Lee S-T, Liu Z (2011). In vivo pharmacokinetics, long-term biodistribution, and toxicology of pegylated graphene in mice. *ACS Nano*.

[68] Von Der Mark K, Park J, Bauer S, Schmuki P (2010). Nanoscale engineering of biomimetic surfaces: cues from the extracellular matrix. *Cell and Tissue Research*.

[69] Allen BL, Kichambare PD, Gou P (2008). Biodegradation of single-walled carbon nanotubes through enzymatic catalysis. *Nano Letters*.

[70] Russier J, Ménard-Moyon C, Venturelli E (2011). Oxidative biodegradation of single- and multi-walled carbon nanotubes. *Nanoscale*.

[71] Kotchey GP, Zhao Y, Kagan VE, Star A (2013). Peroxidase-mediated biodegradation of carbon nanotubes in vitro and in vivo. *Advanced Drug Delivery Reviews*.

[72] Kagan VE, Konduru NV, Feng W (2010). Carbon nanotubes degraded by neutrophil myeloperoxidase induce less pulmonary inflammation. *Nature Nanotechnology*.

[73] Cirillo G, Iemma F, Spizzirri UG (2011). Synthesis of stimuli-responsive microgels for in vitro release of diclofenac diethyl ammonium. *Journal of Biomaterials Science, Polymer Edition*.

[74] Altimari I, Spizzirri UG, Iemma F, Curcio M, Puoci F, Picci N (2012). pH-sensitive drug delivery systems by radical polymerization of gelatin derivatives. *Journal of Applied Polymer Science*.

[75] Li H, Wang DQ, Chen HL, Liu BL, Gao LZ (2003). A novel gelatin-carbon nanotubes hybrid hydrogel. *Macromolecular Bioscience*.

[76] Crommelin DJA, Park K, Florence A (2010). Pharmaceutical nanotechnology: unmet needs in drug delivery. *Journal of Controlled Release*.

[77] Satarkar NS, Johnson D, Marrs B (2010). Hydrogel-MWCNT nanocomposites: synthesis, characterization, and heating with radiofrequency fields. *Journal of Applied Polymer Science*.

[78] Schexnailder P, Schmidt G (2009). Nanocomposite polymer hydrogels. *Colloid and Polymer Science*.

[79] Lovinger AJ (2005). Nano-, bio-, multi-, inter-,...: polymer research in an era of prefixes. *Journal of Macromolecular Science*.

[80] De Heer WA, Bacsa WS, Châtelain A (1995). Aligned carbon nanotube films: production and optical and electronic properties. *Science*.

[81] Park SJ, Lim ST, Cho MS, Kim HM, Joo J, Choi HJ (2005). Electrical properties of multi-walled carbon nanotube/poly(methyl methacrylate) nanocomposite. *Current Applied Physics*.

[82] Aktaş DK, Evingür GA, Pekcan Ö (2010). Critical exponents of gelation and conductivity in polyacrylamide gels doped by multiwalled carbon nanotubes. *Composite Interfaces*.

[83] Hua F, Sun Y, Gaur A (2004). Polymer imprint lithography with molecular-scale resolution. *Nano Letters*.

[84] Wang C, Guo Z-X, Fu S, Wu W, Zhu D (2004). Polymers containing fullerene or carbon nanotube structures. *Progress in Polymer Science*.

[85] Liu I-C, Huang H-M, Chang C-Y, Tsai H-C, Hsu C-H, Tsiang RC-C (2004). Preparing a styrenic polymer composite containing well-dispersed carbon nanotubes: anionic polymerization of a nanotube-bound p-methylstyrene. *Macromolecules*.

[86] Baskaran D, Mays JW, Bratcher MS (2004). Polymer-grafted multiwalled carbon nanotubes through surface-initiated polymerization. *Angewandte Chemie*.

[87] Yan D, Yang G (2009). A novel approach of in situ grafting polyamide 6 to the surface of multi-walled carbon nanotubes. *Materials Letters*.

[88] Mylvaganam K, Zhang LC (2004). Nanotube functionalization and polymer grafting: an ab initio study. *Journal of Physical Chemistry B*.

[89] Gao C, Muthukrishnan S, Li W, Yuan J, Xu Y, Müller AHE (2007). Linear and hyperbranched glycopolymer-functionalized carbon nanotubes: synthesis, kinetics, and characterization. *Macromolecules*.

[90] Li H, Cheng F, Duft AM, Adronov A (2005). Functionalization of single-walled carbon nanotubes with well-defined polystyrene by “click” coupling. *Journal of the American Chemical Society*.

[91] Kong H, Luo P, Gao C, Yan D (2005). Polyelectrolyte-functionalized multiwalled carbon nanotubes: preparation, characterization and layer-by-layer self-assembly. *Polymer*.

[92] Martin RB, Qu L, Lin Y (2004). Functionalized carbon nanotubes with tethered pyrenes: synthesis and photophysical properties. *Journal of Physical Chemistry B*.

[93] Gómez FJ, Chen RJ, Wang D, Waymouth RM, Dai H (2003). Ring opening metathesis polymerization on non-covalently functionalized single-walled carbon nanotubes. *Chemical Communications*.

[94] Cirillo G, Caruso T, Hampel S (2013). Novel carbon nanotube composites by grafting reaction with water-compatible redox initiator system. *Colloid and Polymer Science*.

[95] Lou X, Detrembleur C, Pagnoulle C (2004). Surface modification of multiwalled carbon nanotubes by poly(2-vinylpyridine): dispersion, selective deposition, and decoration of the nanotubes. *Advanced Materials*.

[96] Huang H-M, Liu I-C, Chang C-YU, Tsai H-C, Hsu C-H, Tsiang RC-C (2004). Preparing a polystyrene-functionalized multiple-walled carbon nanotubes via covalently linking acyl chloride functionalities with living polystyryllithium. *Journal of Polymer Science A*.

[97] Qin S, Qin D, Ford WT, Herrera JE, Resasco DE (2004). Grafting of poly(4-vinylpyridine) to single-walled carbon nanotubes and assembly of multilayer films. *Macromolecules*.

[98] Dai X, Liu Z, Han B (2004). Carbon nanotube/poly(2,4-hexadiyne-1,6-diol) nanocomposites prepared with the aid of supercritical CO2. *Chemical Communications*.

[99] Xu H, Wang X, Zhang Y, Liu S (2006). Single-step in situ preparation of polymer-grafted multi-walled carbon nanotube composites under60Co *γ*-ray irradiation. *Chemistry of Materials*.

[100] Petrov P, Lou X, Pagnoulle C, Jérôme C, Calberg C, Jérôme R (2004). Functionalization of multi-walled carbon nanotubes by electrografting of polyacrylonitrile. *Macromolecular Rapid Communications*.

[101] Zeng H, Gao C, Wang Y (2006). In situ polymerization approach to multiwalled carbon nanotubes-reinforced nylon 1010 composites: mechanical properties and crystallization behavior. *Polymer*.

[102] Nogales A, Broza G, Roslaniec Z (2004). Low percolation threshold in nanocomposites based on oxidized single wall carbon nanotubes and poly(butylene terephthalate). *Macromolecules*.

[103] Xu G, Wu W-T, Wang Y (2006). Synthesis and characterization of water-soluble multiwalled carbon nanotubes grafted by a thermoresponsive polymer. *Nanotechnology*.

[104] Cui J, Wang W, You Y, Liu C, Wang P (2004). Functionalization of multiwalled carbon nanotubes by reversible addition fragmentation chain-transfer polymerization. *Polymer*.

[105] Chen S, Chen D, Wu G (2006). Grafting of poly(tBA) and PtBA-b-PMMA onto the surface of SWNTs using carbanions as the initiator. *Macromolecular Rapid Communications*.

[106] Qu L, Veca LM, Lin Y (2005). Soluble nylon-functionalized carbon nanotubes from anionic ring-opening polymerization from nanotube surface. *Macromolecules*.

[107] Buffa F, Hu H, Resasco DE (2005). Side-wall functionalization of single-walled carbon nanotubes with 4-hydroxymethylaniline followed by polymerization of *ε*-caprolactone. *Macromolecules*.

[108] Kong H, Gao C, Yan D (2004). Controlled functionalization of multiwalled carbon nanotubes by in situ atom transfer radical polymerization. *Journal of the American Chemical Society*.

[109] Yao Z, Braidy N, Botton GA, Adronov A (2003). Polymerization from the surface of single-walled carbon nanotubes—preparation and characterization of nanocomposites. *Journal of the American Chemical Society*.

[110] Prakash S, Malhotra M, Shao W, Tomaro-Duchesneau C, Abbasi S (2011). Polymeric nanohybrids and functionalized carbon nanotubes as drug delivery carriers for cancer therapy. *Advanced Drug Delivery Reviews*.

[111] Shi X, Sitharaman B, Pham QP (2007). Fabrication of porous ultra-short single-walled carbon nanotube nanocomposite scaffolds for bone tissue engineering. *Biomaterials*.

[112] Thompson BC, Moulton SE, Gilmore KJ, Higgins MJ, Whitten PG, Wallace GG (2009). Carbon nanotube biogels. *Carbon*.

[113] Song YS (2012). A passive microfluidic valve fabricated from a hydrogel filled with carbon nanotubes. *Carbon*.

[114] Lovat V, Pantarotto D, Lagostena L (2005). Carbon nanotube substrates boost neuronal electrical signaling. *Nano Letters*.

[115] Supronowicz PR, Ajayan PM, Ullmann KR, Arulanandam BP, Metzger DW, Bizios R (2002). Novel current-conducting composite substrates for exposing osteoblasts to alternating current stimulation. *Journal of Biomedical Materials Research*.

[116] Galvan-Garcia P, Keefer EW, Yang F (2007). Robust cell migration and neuronal growth on pristine carbon nanotube sheets and yarns. *Journal of Biomaterials Science, Polymer Edition*.

[117] Abarrategi A, Gutiérrez MC, Moreno-Vicente C (2008). Multiwall carbon nanotube scaffolds for tissue engineering purposes. *Biomaterials*.

[118] Zhang X, Meng L, Lu Q (2009). Cell behaviors on polysaccharide-wrapped single-wall carbon nanotubes: a quantitative study of the surface properties of biomimetic nanofibrous scaffolds. *ACS Nano*.

[119] Zhao B, Hu H, Mandal SK, Haddon RC (2005). A bone mimic based on the self-assembly of hydroxyapatite on chemically functionalized single-walled carbon nanotubes. *Chemistry of Materials*.

[120] Zanello LP, Zhao B, Hu H, Haddon RC (2006). Bone cell proliferation on carbon nanotubes. *Nano Letters*.

[121] Chatterjee S, Lee MW, Woo SH (2009). Enhanced mechanical strength of chitosan hydrogel beads by impregnation with carbon nanotubes. *Carbon*.

[122] Ferris CJ, In Het Panhuis M (2009). Conducting bio-materials based on gellan gum hydrogels. *Soft Matter*.

[123] Puoci F, Hampel S, Parisi OI, Hassan A, Cirillo G, Picci N (2013). I mprinted microspheres doped with carbon nanotubes as novel electroresponsive drug-delivery systems. *Journal of Applied Polymer Science*.

[124] Cirillo G, Vittorio O, Hampel S (2013). Quercetin nanocomposite as novel anticancer therapeutic: improved efficinency and reduced toxicity. *European Journal of Pharmaceutical Sciences*.

[125] Rodrigues AA, Batista NA, Bavaresco VP (2012). In vivo evaluation of hydrogels of polyvinyl alcohol with and without carbon nanoparticles for osteochondral repair. *Carbon*.

[126] Huang Y, Zheng Y, Song W, Ma Y, Wu J, Fan L (2011). Poly(vinyl pyrrolidone) wrapped multi-walled carbon nanotube/poly(vinyl alcohol) composite hydrogels. *Composites Part A*.

[127] Shen X, Chen L, Cai X, Tong T, Tong H, Hu J (2011). A novel method for the fabrication of homogeneous hydroxyapatite/collagen nanocomposite and nanocomposite scaffold with hierarchical porosity. *Journal of Materials Science*.

[128] Calvo-Marzal P, Delaney M, Auletta J (2012). Manipulating mechanical properties with electricity: electroplastic elastomer hydrogels. *ACS Macro Letters*.

[129] Hong SH, Tung TT, Trang LKH, Kim TY, Suh KS (2010). Preparation of single-walled carbon nanotube (SWNT) gel composites using poly(ionic liquids). *Colloid and Polymer Science*.

[130] Zhang X, Liu J, Xu B, Su Y, Luo Y (2011). Ultralight conducting polymer/carbon nanotube composite aerogels. *Carbon*.

[131] Kim K-S, Park S-J (2011). Influence of dispersion of multi-walled carbon nanotubes on the electrochemical performance of PEDOT-PSS films. *Materials Science and Engineering B*.

[132] Xiao Y, He L, Che J (2012). An effective approach for the fabrication of reinforced composite hydrogel engineered with SWNTs, polypyrrole and PEGDA hydrogel. *Journal of Materials Chemistry*.

[133] Saez-Martinez V, Garcia-Gallastegui A, Vera C (2011). New hybrid system: poly(ethylene glycol) hydrogel with covalently bonded pegylated nanotubes. *Journal of Applied Polymer Science*.

[134] Ye Y, Mao Y, Wang H, Ren Z (2012). Hybrid structure of pH-responsive hydrogel and carbon nanotube array with superwettability. *Journal of Materials Chemistry*.

[135] Ogoshi T, Takashima Y, Yamaguchi H, Harada A (2007). Chemically-responsive sol-gel transition of supramolecular single-walled carbon nanotubes (SWNTs) hydrogel made by hybrids of SWNTs and cyclodextrins. *Journal of the American Chemical Society*.

[136] Sui K, Gao S, Wu W, Xia Y (2010). Injectable supramolecular hybrid hydrogels formed by MWNT-grafted- poly(ethylene glycol) and *α*-cyclodextrin. *Journal of Polymer Science A*.

[137] Hui Z, Zhang X, Yu J (2010). Carbon nanotube-hybridized supramolecular hydrogel based on PEO-b-PPO-b-PEO/*α*-cyclodextrin as a potential biomaterial. *Journal of Applied Polymer Science*.

[138] Evingür GA, Pekcan Ö (2012). Monitoring of dynamical processes in PAAmâ€" MWNTs composites by fluorescence method. *Advanced Composite Materials*.

[139] Evingr GA, Pekcan Ö (2012). Elastic percolation of swollen polyacrylamide (PAAm)-multiwall carbon nanotubes composite. *Phase Transitions*.

[140] Zhang X, Pint CL, Lee MH (2011). Optically- and thermally-responsive programmable materials based on carbon nanotube-hydrogel polymer composites. *Nano Letters*.

[141] Cirillo G, Kraemer K, Fuessel S (2010). Biological activity of a gallic acid-gelatin conjugate. *Biomacromolecules*.

[142] Curcio M, Puoci F, Spizzirri UG (2010). Negative thermo-responsive microspheres based on hydrolyzed gelatin as drug delivery device. *AAPS PharmSciTech*.

[143] Cirillo G, Vittorio O, Hampel S, Spizzirri UG, Picci N, Iemma F (2013). Incorporation of carbon nanotubes into a gelatin-catechin conjugate: innovative approach for the preparation of anticancer materials. *International Journal of Pharmaceutics*.

[144] Haider S, Park S-Y, Saeed K, Farmer BL (2007). Swelling and electroresponsive characteristics of gelatin immobilized onto multi-walled carbon nanotubes. *Sensors and Actuators B*.

[145] Spizzirri UG, Hampel S, Cirillo G (2013). Spherical gelatin/CNTs hybrid microgels as electro-responsive drug delivery systems. *International Journal of Pharmaceutics*.

[146] Roy S, Banerjee A (2012). Functionalized single walled carbon nanotube containing amino acid based hydrogel: a hybrid nanomaterial. *RSC Advances*.

[147] Yun J, Im JS, Lee Y-S, Kim H-I (2011). Electro-responsive transdermal drug delivery behavior of PVA/PAA/MWCNT nanofibers. *European Polymer Journal*.

[148] Im JS, Bai BC, Lee Y-S (2010). The effect of carbon nanotubes on drug delivery in an electro-sensitive transdermal drug delivery system. *Biomaterials*.

[149] Servant A, Methven L, Williams RP, Kostarelos K (2013). Electroresponsive polymer-carbon nanotube hydrogel hybrids for pulsatile drug delivery in vivo. *Advanced Healthcare Materials*.

[150] Li C, Mezzenga R (2012). Functionalization of multiwalled carbon nanotubes and their pH-responsive hydrogels with amyloid fibrils. *Langmuir*.

[151] Cheng E, Li Y, Yang Z, Deng Z, Liu D (2011). DNA-SWNT hybrid hydrogel. *Chemical Communications*.

[152] Kovtyukhova NI, Mallouk TE, Pan L, Dickey EC (2003). Individual single-walled nanotubes and hydrogels made by oxidative exfoliation of carbon nanotube ropes. *Journal of the American Chemical Society*.

[153] Sui Z, Meng Q, Zhang X, Ma R, Cao B (2012). Green synthesis of carbon nanotube-graphene hybrid aerogels and their use as versatile agents for water purification. *Journal of Materials Chemistry*.

